# Supplementary material for: Genome-Wide Analysis of the MADS-Box Gene Family in Hibiscus syriacus and Their Role in Floral Organ Development
Source: Int J Mol Sci. 2023 Dec 28;25(1):406. doi: 10.3390/ijms25010406 (PMC10779063; doi:10.3390/ijms25010406)
Supplement: Supplementary file 1 [file ijms-25-00406-s001.zip › Supplement Table S1.pdf]

Supplement Table S1. The MADS-box genes identified in *H. syriacus*

Serial no.	Gene ID	Gene name	PI	MW (KDa)	A.A	Group
1	rna-XM_039135021.1	*HsMADS1*	9.75	26639.36	234	MIKC
2	rna-XM_039135281.1	*HsMADS2*	9.08	24035.46	208	MIKC
3	rna-XM_039136060.1	*HsMADS3*	9.12	28677.02	252	Mγ
4	rna-XM_039136443.1	*HsMADS4*	8.79	28345.19	249	MIKC
5	rna-XM_039136654.1	*HsMADS5*	6.11	28246.81	248	MIKC
6	rna-XM_039136804.1	*HsMADS6*	6.84	27553.53	243	Mγ
7	rna-XM_039138197.1	*HsMADS7*	7.69	28286.08	247	MIKC
8	rna-XM_039138310.1	*HsMADS8*	8.26	28632.48	247	MIKC
9	rna-XM_039138568.1	*HsMADS9*	9.33	25454.12	221	MIKC
10	rna-XM_039138569.1	*HsMADS10*	9.33	25454.12	221	MIKC
11	rna-XM_039138795.1	*HsMADS11*	6.19	18007.58	156	Mγ
12	rna-XM_039140029.1	*HsMADS12*	9.42	25930.58	223	MIKC
13	rna-XM_039140030.1	*HsMADS13*	9.75	16923.62	147	MIKC
14	rna-XM_039140539.1	*HsMADS14*	9.51	27209.82	241	MIKC
15	rna-XM_039141146.1	*HsMADS15*	8.99	29246.25	254	MIKC
16	rna-XM_039142382.1	*HsMADS16*	10.15	12314.58	108	MIKC
17	rna-XM_039142747.1	*HsMADS17*	8.57	28383.18	249	MIKC
18	rna-XM_039143452.1	*HsMADS18*	9.16	27781.78	241	MIKC
19	rna-XM_039143574.1	*HsMADS19*	8.58	27931.76	245	MIKC
20	rna-XM_039145139.1	*HsMADS20*	8.71	28030.87	245	MIKC
21	rna-XM_039146181.1	*HsMADS21*	8.67	30983.3	273	MIKC
22	rna-XM_039146182.1	*HsMADS22*	8.83	30726.06	271	MIKC
23	rna-XM_039147373.1	*HsMADS23*	5.98	27644.58	243	Mγ
24	rna-XM_039147868.1	*HsMADS24*	9.21	28336.04	246	Mδ
25	rna-XM_039148029.1	*HsMADS25*	9.54	25226.8	215	MIKC
26	rna-XM_039148030.1	*HsMADS26*	9.54	25226.8	215	MIKC
27	rna-XM_039148256.1	*HsMADS27*	9.08	24781.39	217	MIKC
28	rna-XM_039148257.1	*HsMADS28*	9.08	24781.39	217	MIKC
29	rna-XM_039148258.1	*HsMADS29*	9.08	24653.26	216	MIKC
30	rna-XM_039148290.1	*HsMADS30*	5.4	39560.4	368	Mβ
31	rna-XM_039149633.1	*HsMADS31*	9.8	23953.05	209	MIKC
32	rna-XM_039152162.1	*HsMADS32*	9.75	20235.38	174	MIKC
33	rna-XM_039152207.1	*HsMADS33*	8.79	27877.83	246	MIKC
34	rna-XM_039152214.1	*HsMADS34*	9.45	25248.18	217	Mα
35	rna-XM_039152215.1	*HsMADS35*	9.45	25248.18	217	MIKC
36	rna-XM_039153148.1	*HsMADS36*	9.31	28812.71	250	MIKC
37	rna-XM_039153628.1	*HsMADS37*	8.81	28107.09	245	MIKC

38	rna-XM_039153695.1	*HsMADS38*	9.02	24845.52	213	MIKC
39	rna-XM_039155415.1	*HsMADS39*	8.89	23296.85	201	MIKC
40	rna-XM_039155416.1	*HsMADS40*	8.89	23296.85	201	MIKC
41	rna-XM_039155568.1	*HsMADS41*	9.91	24294.83	209	MIKC
42	rna-XM_039155574.1	*HsMADS42*	9.28	25175.77	219	MIKC
43	rna-XM_039155575.1	*HsMADS43*	9.05	23445.73	205	MIKC
44	rna-XM_039156291.1	*HsMADS44*	8.39	14710.23	132	MIKC
45	rna-XM_039157544.1	*HsMADS45*	6.63	28770.5	253	MIKC
46	rna-XM_039158351.1	*HsMADS46*	9.48	24976.57	222	Mα
47	rna-XM_039158843.1	*HsMADS47*	9.02	25525.03	219	Mδ
48	rna-XM_039160748.1	*HsMADS48*	9.56	19276.32	169	MIKC
49	rna-XM_039161036.1	*HsMADS49*	8.84	24732.24	217	MIKC
50	rna-XM_039161043.1	*HsMADS50*	8.84	24732.24	217	MIKC
51	rna-XM_039161048.1	*HsMADS51*	8.84	24732.24	217	MIKC
52	rna-XM_039161056.1	*HsMADS52*	8.84	24604.11	216	MIKC
53	rna-XM_039162021.1	*HsMADS53*	8.86	27981.83	245	MIKC
54	rna-XM_039162265.1	*HsMADS54*	8.57	28292.13	247	MIKC
55	rna-XM_039162948.1	*HsMADS55*	9.28	29486.93	260	Mγ
56	rna-XM_039162997.1	*HsMADS56*	8.58	28170.97	249	MIKC
57	rna-XM_039162998.1	*HsMADS57*	8.58	28170.97	249	MIKC
58	rna-XM_039163273.1	*HsMADS58*	4.88	25503.41	231	Mα
59	rna-XM_039163317.1	*HsMADS59*	4.88	25534.43	231	Mα
60	rna-XM_039164292.1	*HsMADS60*	9.49	28228.22	245	MIKC
61	rna-XM_039164293.1	*HsMADS61*	9.43	28000.91	243	MIKC
62	rna-XM_039164645.1	*HsMADS62*	8.51	27970.75	245	MIKC
63	rna-XM_039164838.1	*HsMADS63*	8.64	25752.39	222	MIKC
64	rna-XM_039165917.1	*HsMADS64*	9.87	27376.16	241	MIKC
65	rna-XM_039166059.1	*HsMADS65*	9.32	27699.65	240	MIKC
66	rna-XM_039166060.1	*HsMADS66*	9.19	27543.46	239	MIKC
67	rna-XM_039166061.1	*HsMADS67*	9.25	25939.56	226	MIKC
68	rna-XM_039166063.1	*HsMADS68*	9.94	16244.79	142	MIKC
69	rna-XM_039166802.1	*HsMADS69*	9.17	24443.81	213	MIKC
70	rna-XM_039167230.1	*HsMADS70*	6.45	23865.98	207	MIKC
71	rna-XM_039167371.1	*HsMADS71*	8.81	25268.91	220	MIKC
72	rna-XM_039167859.1	*HsMADS72*	8.67	24266.69	217	Mα
73	rna-XM_039168616.1	*HsMADS73*	9.55	25393.13	221	MIKC
74	rna-XM_039169411.1	*HsMADS74*	6.87	27425.07	239	MIKC
75	rna-XM_039171324.1	*HsMADS75*	8.96	28200.24	246	MIKC
76	rna-XM_039172290.1	*HsMADS76*	6.84	22589.54	199	MIKC
77	rna-XM_039172291.1	*HsMADS77*	6.84	22621.6	199	MIKC
78	rna-XM_039172292.1	*HsMADS78*	6.84	21086.73	185	MIKC

79	rna-XM_039172293.1	*HsMADS79*	6.84	21118.79	185	MIKC
80	rna-XM_039173054.1	*HsMADS80*	7.7	28683.67	246	MIKC
81	rna-XM_039173451.1	*HsMADS81*	8.78	24979.71	214	MIKC
82	rna-XM_039173869.1	*HsMADS82*	8.89	28045.87	245	MIKC
83	rna-XM_039174006.1	*HsMADS83*	8.15	32656.31	286	MIKC
84	rna-XM_039174007.1	*HsMADS84*	8.87	29095.26	256	MIKC
85	rna-XM_039174161.1	*HsMADS85*	8.96	26799.67	240	Mα
86	rna-XM_039174618.1	*HsMADS86*	9.11	28039.86	243	MIKC
87	rna-XM_039175788.1	*HsMADS87*	8.8	46384.13	409	Mβ
88	rna-XM_039178309.1	*HsMADS88*	8.58	27790.58	245	MIKC
89	rna-XM_039178739.1	*HsMADS89*	7.66	28190.99	244	MIKC
90	rna-XM_039179203.1	*HsMADS90*	8.58	26700.55	240	Mα
91	rna-XM_039179413.1	*HsMADS91*	9.55	18125.79	157	MIKC
92	rna-XM_039179415.1	*HsMADS92*	9.75	16519.97	143	MIKC
93	rna-XM_039180445.1	*HsMADS93*	9.28	24543.13	213	MIKC
94	rna-XM_039181975.1	*HsMADS94*	8.73	26304.14	236	MIKC
95	rna-XM_039182102.1	*HsMADS95*	9.81	21968.38	187	MIKC
96	rna-XM_039182695.1	*HsMADS96*	9.24	26831.47	232	MIKC
97	rna-XM_039183245.1	*HsMADS97*	8.91	28362.46	245	MIKC
98	rna-XM_039184009.1	*HsMADS98*	9.06	26204.89	234	Mα
99	rna-XM_039184190.1	*HsMADS99*	9.35	24142.72	207	MIKC
100	rna-XM_039185159.1	*HsMADS100*	8.78	28245.12	249	MIKC
101	rna-XM_039185529.1	*HsMADS101*	9.86	13378.35	119	MIKC
102	rna-XM_039185995.1	*HsMADS102*	9.51	25394.11	220	MIKC
103	rna-XM_039186099.1	*HsMADS103*	5.25	18344.04	159	Mγ
104	rna-XM_039186608.1	*HsMADS104*	9.23	25015.95	217	MIKC
105	rna-XM_039186609.1	*HsMADS105*	9.36	24783.63	217	MIKC
106	rna-XM_039186732.1	*HsMADS106*	9.3	23260.57	203	MIKC
107	rna-XM_039187971.1	*HsMADS107*	8.57	28383.18	249	MIKC
108	rna-XM_039188392.1	*HsMADS108*	9.79	25884.58	226	MIKC
109	rna-XM_039188917.1	*HsMADS109*	9.6	16571.2	143	MIKC
110	rna-XM_039189007.1	*HsMADS110*	9.41	39363.4	343	Mβ
111	rna-XM_039191480.1	*HsMADS111*	9.06	30178.47	260	MIKC
112	rna-XM_039191481.1	*HsMADS112*	9.09	26811.48	232	MIKC
113	rna-XM_039192059.1	*HsMADS113*	6.97	27845.88	242	MIKC
114	rna-XM_039192379.1	*HsMADS114*	9.53	28120.9	241	MIKC
115	rna-XM_039193599.1	*HsMADS115*	9.34	22478.08	197	Mα
116	rna-XM_039194062.1	*HsMADS116*	9.44	25732.51	225	MIKC
117	rna-XM_039194063.1	*HsMADS117*	9.44	25732.51	225	MIKC
118	rna-XM_039194064.1	*HsMADS118*	9.41	20093.03	175	MIKC
119	rna-XM_039194246.1	*HsMADS119*	8.34	24770.3	214	MIKC

120	rna-XM_039194542.1	*HsMADS120*	9.12	25747.3	225	MIKC
121	rna-XM_039195651.1	*HsMADS121*	8.99	24720.34	217	MIKC
122	rna-XM_039195652.1	*HsMADS122*	8.99	24592.21	216	MIKC
123	rna-XM_039196974.1	*HsMADS123*	9.32	24733.17	222	Mα
124	rna-XM_039197641.1	*HsMADS124*	9.47	26796.57	232	MIKC
125	rna-XM_039198777.1	*HsMADS125*	8.56	28322.21	245	MIKC
126	rna-XM_039198778.1	*HsMADS126*	9.43	26160.73	224	MIKC
127	rna-XM_039199108.1	*HsMADS127*	8.76	28150.02	243	MIKC
128	rna-XM_039199623.1	*HsMADS128*	6.66	24043.59	217	MIKC
129	rna-XM_039200913.1	*HsMADS129*	9.4	31858.78	278	Mβ
130	rna-XM_039200963.1	*HsMADS130*	4.99	25157.17	226	Mα
131	rna-XM_039202446.1	*HsMADS131*	5.64	48866.5	447	Mβ
132	rna-XM_039202497.1	*HsMADS132*	8.53	24635.96	225	Mα
133	rna-XM_039202677.1	*HsMADS133*	9.65	22300.5	190	MIKC
134	rna-XM_039202909.1	*HsMADS134*	9.38	11445.19	100	MIKC
135	rna-XM_039203255.1	*HsMADS135*	7.13	28052.77	248	MIKC
136	rna-XM_039203448.1	*HsMADS136*	5.33	40074.72	350	Mδ
137	rna-XM_039205223.1	*HsMADS137*	5.02	23965	214	Mα
138	rna-XM_039206724.1	*HsMADS138*	6.97	25356.88	219	MIKC
139	rna-XM_039207035.1	*HsMADS139*	8.72	26160.82	233	Mα
140	rna-XM_039208530.1	*HsMADS140*	9.47	23239.83	201	MIKC
141	rna-XM_039208531.1	*HsMADS141*	9.47	23239.83	201	MIKC
142	rna-XM_039208554.1	*HsMADS142*	8.55	28460.33	247	MIKC
143	rna-XM_039209158.1	*HsMADS143*	9.3	28067.72	243	MIKC
144	rna-XM_039209159.1	*HsMADS144*	9.3	28067.72	243	MIKC
145	rna-XM_039209503.1	*HsMADS145*	8.6	27830.56	245	MIKC
146	rna-XM_039211675.1	*HsMADS146*	8.38	22299.48	194	MIKC
147	rna-XM_039212525.1	*HsMADS147*	9.04	26641.41	237	Mα
148	rna-XM_039212714.1	*HsMADS148*	9.3	27197.17	236	MIKC
149	rna-XM_039212715.1	*HsMADS149*	9.3	27197.17	236	MIKC
150	rna-XM_039212716.1	*HsMADS150*	9.52	25515.28	222	MIKC
151	rna-XM_039213056.1	*HsMADS151*	8.8	27869.68	245	MIKC
152	rna-XM_039214344.1	*HsMADS152*	8.69	28224.36	250	MIKC
153	rna-XM_039214345.1	*HsMADS153*	8.69	28096.23	249	MIKC
154	rna-XM_039214346.1	*HsMADS154*	8.84	24732.24	217	MIKC
155	rna-XM_039214347.1	*HsMADS155*	8.84	24732.24	217	MIKC
156	rna-XM_039214348.1	*HsMADS156*	8.84	24732.24	217	MIKC
157	rna-XM_039214761.1	*HsMADS157*	9.95	20113.49	179	Mα
158	rna-XM_039214790.1	*HsMADS158*	9.4	27691.42	239	MIKC
159	rna-XM_039214791.1	*HsMADS159*	9.4	27691.42	239	MIKC
160	rna-XM_039214802.1	*HsMADS160*	10.18	20383.65	182	Mα

161	rna-XM_039215063.1	HsMADS161	9.53	27510.29	239	MIKC
162	rna-XM_039215365.1	HsMADS162	5.69	40052.69	350	Mδ
163	rna-XM_039215811.1	HsMADS163	9.14	46340.97	409	Mβ

Supplement Table S2. FPKM values of 9 HsMADS-box genes

Gene ID	S1-0.3	S2-0.3	S3-0.3	S1-0.7	S2-0.7	S3-0.7	D1-0.3	D2-0.3	D3-0.3	D1-0.7	D2-0.7	D3-0.7
HsMADS48	9.549	12.9578	8.4497	10.0223	12.8621	12.3892	0.4319	0.6673	0.5413	1.5306	1.2511	1.9522
HsMADS145	7.8055	7.6013	7.3861	18.4519	13.378	14.944	23.8467	26.6283	23.3034	56.2816	46.6288	55.9479
HsMADS111	5.6085	6.277	4.2563	10.7669	11.2856	9.4294	0.8764	1.1733	0.2799	1.1589	1.3357	2.4426
HsMADS8	24.9729	31.3997	31.5295	38.3612	30.9139	29.5442	57.7636	64.9391	49.1202	77.9837	74.6492	88.4042
HsMADS33	13.7943	15.3989	13.0874	15.0479	15.0381	14.4422	33.8514	26.8323	26.7764	35.2002	29.5686	40.3495
HsMADS161	20.4975	22.5779	17.6987	16.2709	15.8445	18.8753	8.6987	10.239	7.5301	7.5954	7.7853	7.9949
HsMADS138	198.0667	159.3854	110.1611	257.9341	246.937	248.5955	102.2927	88.3573	100.3062	229.2853	209.5436	254.8195
HsMADS143	6.9131	8.484	4.7816	11.89	8.1312	11.3223	1.0905	1.9635	1.1531	4.6965	4.086	4.0449
HsMADS69	2.4861	1.8464	1.5258	3.8294	3.3316	4.2142	0.2323	0.6996	0.5787	1.6643	1.0494	1.0077

The S represent sing flower and the D represent double flower. The 1, 2, 3 represent biological replicates. The 0.3 and 0.7 represent different flower bud lengths (0.3 cm and 0.7 cm).

Supplement Table S3. Primers used for qRT-PCR.

Gene name	Gene ID	Forward primer	Reverse primer
HsMADS8	LOC120118188	TGATCGTCTTCTCCCACAAAG	GCTGTCTCTCTGCATACGAAT
HsMADS33	LOC120136096	AGTGGAGCTGAAGAGGATAGA	ACGGCTGGAGAAGACAATAAG
HsMADS48	LOC120147348	CCGACAACTACGCAATCAAATC	ACGAGACTCCAAGCTCTTTAAC
HsMADS69	LOC120155226	GCTTATGGAATCAGCAGCAAAG	CTAGTCCATGTCCCAGCATTT
HsMADS111	LOC120187877	CGTTGCTGAAGCCAACATTC	CTCTCCAAGGATATGCCTGTTC
HsMADS138	LOC120207201	GAGGATATCTCATCTCTGCCTTAC	CCTTGCCACATCAAGAACATC
HsMADS143	LOC120210443	AGGTCGCTTTGATCGTCTTC	CTTCTGCAACTGACCCAGTAT
HsMADS145	LOC120210837	GCGTATGAGCTTTCGGTTCT	CACTTCTGGTACCTCTCCAATG
HsMADS161	LOC120218081	CCAGATCAAGCGGATCGAAA	GGCAACTTCAGCATCACAAAG
