# Supplementary figures and images for: Genome-Wide Analysis of the MADS-Box Gene Family in Hibiscus syriacus and Their Role in Floral Organ Development
Source: Int J Mol Sci. 2023 Dec 28;25(1):406. doi: 10.3390/ijms25010406 (PMC10779063; doi:10.3390/ijms25010406)

**Supplement Figure S1.** Details of multiple sequence alignment of 163 HsMADS proteins

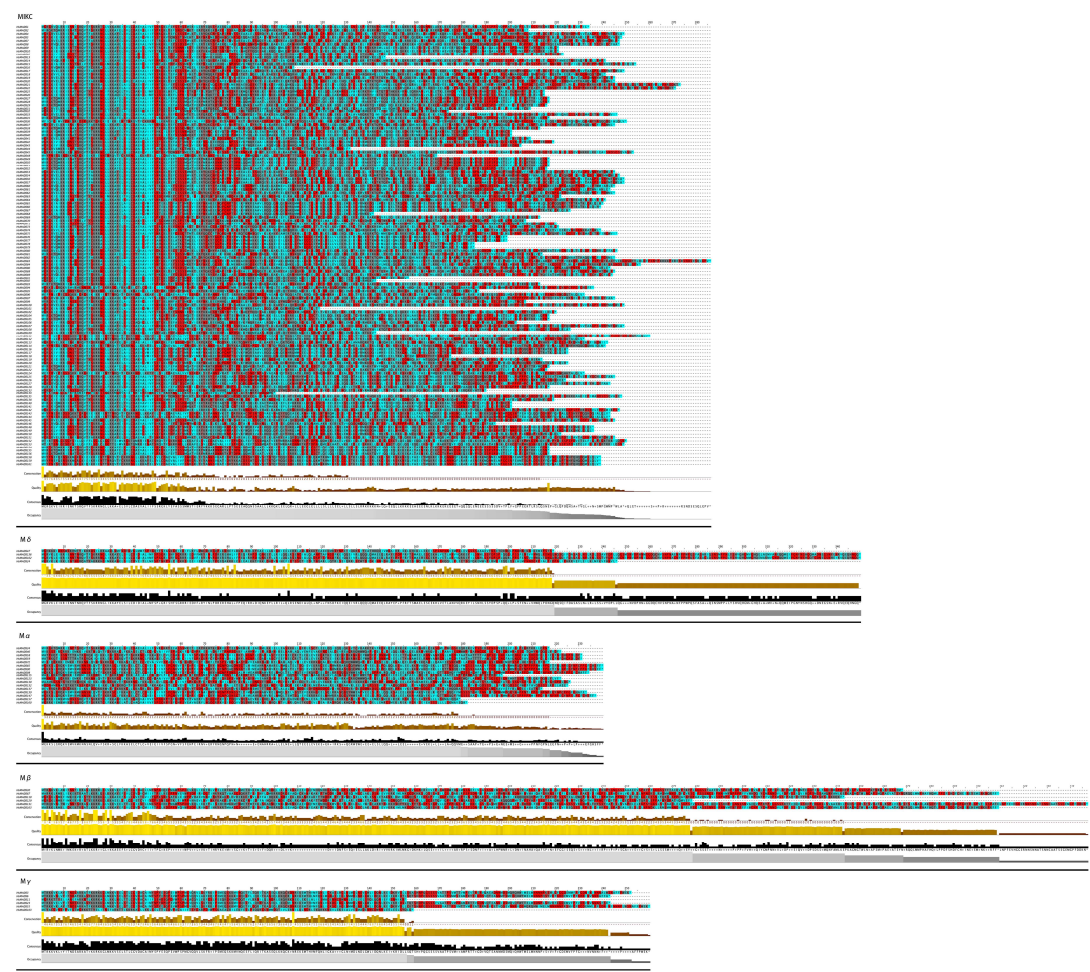

Supplement: Supplementary file 1 [file ijms-25-00406-s001.zip › Supplement Figure S1.pdf]
